# The consent process: Enabling or disabling patients’ active participation?

**DOI:** 10.1177/1363459315611870

**Published:** 2015-10-20

**Authors:** Carole Doherty, Charitini Stavropoulou, Mark NK Saunders, Tracey Brown

**Affiliations:** University of Surrey, UK; City University London, UK; Birmingham University, UK; Dallas McMillan Solicitors, UK

**Keywords:** caregivers, consent, patients, UK National Health Service

## Abstract

Standards expected by doctors’ regulatory bodies in respect of the process of consent to treatment have arguably sought to restructure the nature of the doctor–patient relationship from one of the paternalism to that of shared decision-making. Yet, few studies have explored empirically, from patients’ perspectives, the extent to which the process of consent to treatment enables or disables patients’ participation in medical decision-making. Our article examines patients’ attitudes towards the consent process, exploring how and why these attitudes influence patients’ active participation in decision-making and considering possible consequent medico-legal issues. Data were collected longitudinally using semi-structured interviews and field observations involving 35 patients and 19 of their caregivers, in an English hospital between February and November 2014. These indicate that generally patients defer to the doctor in respect of treatment decision-making. Although most patients and their caregivers wanted detailed information and discussion, they did not necessarily expect that this would be provided. Furthermore, patients perceived that signing the consent form was an obligatory routine principally to protect doctors from legal action should something go wrong. Our study suggests that patients’ predominantly paternalistic perceptions of the consent process can not only undermine attempts by doctors to involve them in decision-making but, as patients are now considered in law as informed actors, their perceptions of the consent form as not being in their interests could be a self-fulfilling prophecy if signing is undertaken without due consideration to the content.

## Introduction

Standards expected by doctors’ regulatory bodies in respect of the consent process have arguably sought to restructure the nature of the doctor–patient relationship from paternalism to shared decision-making (AMA, 2009; Charles et al., 1999; Fovargue and Miola, 2010; GMC, 2008; World Medical Association (WMA), 2006). Nonetheless, a substantial gap remains between the practice of informed consent and its intended goals (Grady, 2015). Studies of consent have tended to examine consent to research (Gammelgaard et al., 2004; Wade et al., 2009), patients’ understanding of risk (Ashraff et al., 2006; Garcia-Retamero and Galesic, 2010), the quality of the consent process (Habiba et al., 2004) and patients’ understandings of written consent and the information provided (Akkad et al., 2006; Fink et al., 2010; Mulsow et al., 2012).

Patients’ attitudes and beliefs about the purpose of consent invariably influence how the process is co-constructed in practice. Examining patients’ and their caregivers’ assumptions and beliefs about the consent process in the United Kingdom, our article responds to a call for further research on consent, involving patients and their caregivers, to help guide clinical practice and bridge the gap between the theory and practice of informed consent (Grady, 2015). We therefore explore how and why patients’ attitudes towards the consent process influence their active participation in decision-making and consider the possible consequent medico-legal issues. This research is timely as UK law has changed recently from judgements based on the reasonable practitioner standard to that of a reasonable person in the patient’s position (*Montgomery v Lanarkshire Health Board*, 2015), this being closer to the medical profession’s (espoused) standard.

Over the past three decades, there has been gradual movement towards reform of the doctor–patient relationship from one designed to sustain doctors’ professional autonomy and benign paternalism (Kaufmann, 1983), to one promoting patient autonomy and shared decision-making (Kon, 2010). The Western philosophical belief of ‘autonomy’ or self-determination emphasises respect for individuals’ rights to control their lives and actions by their own choices, ‘at least to the greatest extent compatible with the rights of others’ (Herbert, 1980: 1042). Medical paternalism, the belief stated simply that ‘doctor knows best’, has been tested by a number of factors. These include the complexity and range of treatment options; appreciation that it is the patient, not the doctor, who has to live with the consequences of decisions made; and the recognition that doctors do not always know what is current best practice (Charles et al., 1999).

However, and although there has been an increased advocacy for shared decision-making, a study involving adults in the United States found that there was still significant paternalism in medical practice (Fowler et al., 2013). Similarly, a recent inquiry into the practice of an English surgeon performing a variation of a mastectomy unrecognised by his peers, argued that if

the prevailing culture is one in which the patient is seen as the recipient of whatever is on offer, then consent can come to be seen as some perfunctory exercise … Hence, the patient is ‘consented’ and the doctor can then get on with things, having had to pause as briefly as possible to tick the consent box. (Kennedy, 2013: 52)

We commence by discussing the concept of informed consent from both a medical and a legal perspective. We then outline our method. Subsequently, we present findings based on a longitudinal qualitative study of the consent process from the perspective of patients (and their caregivers) undergoing treatment for cancer. We conclude by discussing the implications of our study for patients’ involvement as active partners in clinical decision-making.

## Consent to treatment

In the UK National Health Service (NHS), as in many other countries, the consent to treatment process is grounded in the principle of the individual’s right to self-determination over her or his own body. For adults, consent is considered valid when it is given voluntarily by an individual capable of understanding the information provided and able to make a decision based on their evaluation of the risks and benefits (DH, 2009). The principle of documenting patients’ consent was established originally as a legal instrument for extending the liability of doctors in the event of harm arising from clinical care. Consent comprises of the concepts of ‘battery’ and of ‘negligence’. Battery is touching a patient without effective consent either implied or explicit, which may constitute a civil or criminal offence (DH, 2009). Negligence is failure to provide the patient with all necessary information about the procedure including significant possible adverse outcomes and the patient subsequently suffers harm as a result of treatment (DH, 2009). Although the patient’s signature on a (consent) form does not in itself constitute valid consent and is generally not a legal requirement, such forms are often considered good practice where an intervention such as surgery is proposed (DH, 2009).

Doctors’ regulatory bodies emphasise the relationship between doctors and patients focusing on building trust, openness and good communications (AMA, 2009; GMC, 2008). UK current guidance states ‘Each person has a role to play in making decisions about treatment or care’ (GMC, 2008: 7) before listing the complementary responsibilities of those involved. Rather than something a doctor does to a patient, consent is considered a two-way process in which the patient is (or should be) the active partner in giving consent to treatment (Kennedy, 2013; Sokol, 2014). Although the patient requires sufficient information to make a decision, the doctor should also be checking the patient has understood what is proposed and the associated risk (DH, 2009; *Montgomery v Lanarkshire Health Board*, 2015). The patient has the right to refuse detailed information but, as preferences can change over time, consent to treatment should be an on-going process for the duration of the person’s treatment (DH, 2009). Nonetheless, the process of achieving informed consent is displayed most clearly as concluding when the patient signs a consent form (Kinnersley et al., 2013).

Where harm has occurred and the courts become involved, UK judgements have tended to be made on what is deemed to be the appropriate standards of disclosure of the risks involved (Jones, 1999). Standards of disclosure relate to the doctor’s legal duty, rather than the patient’s need for, or understanding of, information. They are therefore an issue of medico-legal practice rather than the patient’s right to decision-making (Fovargue and Miola, 2010; Jones, 1999). It has been judged that, ‘the provision of too much information may prejudice the attainment of the objective of restoring the patient’s health’ (*Sidaway v. Board of Governors of the Bethlem Royal Hospital*, 1985: 904). Consequently, the ‘responsible practitioner standard’ has tended to take precedence. This requires the doctor involved to demonstrate that he or she provided information to the patient that a doctor of good standing in the doctor’s community of peers would provide to her or his patient (*Bolam v Friern Hospital Management Committee*, 1957).

Within such judgements, the importance of patient autonomy has been recognised increasingly by the courts (see e.g. *Chester v Afshar*, 2004). A recent Supreme Court ruling (*Montgomery v Lanarkshire Health Board*, 2015) judged that a risk is material if a reasonable person in the patient’s position would be likely to attach significance to it, or if the doctor is or should reasonably be aware that *their* patient would be likely to attach significance to it. The ruling stated that ‘There is no reason to perpetuate the application of the *Bolam* test’ (p. 28). This ruling places greater emphasis on the ethical principle of patient autonomy, being closer to current General Medical Council (GMC) guidance, and practice in parts of the United States, Canada and Australia (Jones, 1999; *Reibl v Hughes*, 1980; *Rogers v Whitaker*, 1992).

Nonetheless, such patient autonomy is not without problems. In a study involving cancer patients in Canada, Sinding et al. (2010) found that patients were uncomfortable with what they perceived as an expectation that they were responsible for making decisions about their treatment. When patients are left to decide on the type of treatment, they may feel unsupported through having to make decisions based on information that they might not fully understand (Fovargue and Miola, 2010; Sinding et al., 2010). This supports an argument that requirements to provide patients with more information are being conflated with the informed model, that is where the patient makes the decision, rather than the shared decision-making model (Charles et al., 1999; Fovargue and Miola, 2010; Jones, 1999).

Arguably, the apparent contractual nature of the consent form is not a natural aspect of the doctor–patient relationship and can act as a barrier to effective dialogue (Clements, 2005; Meredith, 1993). This may be especially problematic if, as studies suggest, doctor–patient interaction tends to be dominated by doctors’ biomedical agenda with little time given to patients’ functional or emotional concerns (Pilnick and Dingwall, 2011). If patients are to be active participants in decision-making, then they need to believe that their concerns are important and appreciated (Arora, 2003) and to have the opportunity to build relationships or ‘get to know’ their doctors (Hor et al., 2013). Evidence indicates that patients’ understanding is maximised where the consent process takes between 15 and 30 minutes (Fink et al., 2010). However, there is a perception held by some patients of the consent process not being in their interests. Rather it is a legal document designed to protect the rights of doctors and legitimise their decision-making, therefore being of less relevance to patients (Akkad et al., 2004; Cassileth et al., 1980; Habiba et al., 2004).

Patients holding such assumptions and beliefs about consent to treatment and their role within that process will invariably influence its co-construction in practice. Consequently, this study examines, from patients’ and their caregivers’ perspectives, how and why their attitudes to consent influence their participation in decision-making and considers the possible consequent medico-legal issues.

## Method

### Research setting

Data were collected from a sample of day-case patients and their caregivers (a patient’s relative or close friend) over the period of their chemotherapy treatment, in an English tertiary cancer treatment centre between February and November 2014. This centre had been rated more highly than most NHS trusts in recent patient surveys. In 2014, it scored ‘above average’ for eight of the nine aspects of in-patient experience and, in 2012, ‘above average’ for seven of the nine aspects of out-patient care and treatments (Care Quality Commission, 2014). The research site provided access to patients with experience of primary, secondary and tertiary healthcare, many having consented to surgery before being referred to this hospital for chemotherapy. Issues of informed consent are particularly important for such people diagnosed with cancer, and their caregivers, because the disease is potentially life threatening, and therefore life changing, surgery can be complex and the extent of the procedure(s) may not always be known in advance. In addition, chemotherapy treatment may have many side effects such as vomiting and decreased immunity with risk of death or serious illness from infections.

### Research design

We were concerned with both patients and their caregivers understanding of the consent form, including how actively involved they were in the process of consent to treatment. Previous research (Doherty and Saunders, 2013) had highlighted that obtaining patients’ narratives about their experiences at specified stages in their treatment could allow chronological connections and temporal sequencing of events to be explored (De Fina and Georgakopoulou, 2008), identifying possible changes in inter-subjective experiences (Syrjälä et al., 2009). We therefore adopted a longitudinal- and processual-based research design to construct inductively thick descriptions (Geertz, 1973) of patients’ and their caregivers’ narratives of their treatment experiences. Having obtained NHS Research and Ethics approval, data were collected using initial, mid-way through and post-chemotherapy cycle narrative interviews supplemented by field observations.

A maximum variation purposive sample (Saunders, 2012) of 35 patients (17 males and 18 females) aged between 34 and 82 years was selected. Their mean age of 62 was similar to that of UK cancer patients, the majority of whom are aged over 60 years (Cancer Research UK, 2015). We sought to ensure that our patient participants included people with a variety of cancers including breast, bladder, lung and bowel, thereby allowing key themes across a range of treatments to be revealed. We also sought to select patient participants of varying ages, any patterns that emerged being likely to be of wider interest and value (Patton, 2002). In all, 19 of their caregivers (7 men and 12 women) also agreed to participate, following agreement to this by the respective patients. Although few guidelines are available for estimating sample size for qualitative research, the majority of recommendations fall within a range of 10–50 participants (Saunders, 2012). More specifically, research indicates that to achieve data saturation in qualitative interviews with patients and their relatives, between 10 and 15 participants are likely to be required (Francis et al., 2010). Overall, our sample of 35 patients and 19 of their caregivers therefore falls within these recommendations.

Each participant was identified by a code comprising whether they were a patient (P) or caregiver (C), their gender (M = male, F = female) and age in years. Participants of the same code were differentiated by a third alphabetic character, a note being made to enable patients and their caregivers to be linked. Although 35 patients and 19 caregivers consented to participate, there was invariably attrition, six patients and eight caregivers being unable or declining to take part in at least one of the subsequent interviews (Table 1).

**Table 1.** Participant interviews.

Patient participants			Number of interviews per patient	Number of patients	Total number of interviews
Therapy stage at which interview conducted					
Initial	Mid-way	Post			
✓	✓	✓	N = 3	N = 29	N = 87
✓	✓	×	N = 2	N = 3	N = 5
✓	×	×	N = 1	N = 3	N = 3
**Total**				**N** **=** **35**	**N** **=** **95**
Caregiver participants			Number of interviews per caregiver	Number of caregivers	Total number of interviews
Therapy stage at which interview conducted					
Initial	Mid-way	Post			
✓	✓	✓	N = 3	N = 11	N = 33
✓	×	✓	N = 2	N = 4	N = 8
✓	×	×	N = 1	N = 4	N = 4
Total				N = 19	N = 45

Interviews were conducted and audio-recorded in a private office in the hospital, patients being present at four of their caregivers’ interviews. Both patient and caregiver participants were asked to talk about their experience of cancer diagnosis and treatment, what they considered the purpose of consent and the form, and whether the patient had been asked to sign this form. Interview questions were deliberately broad and open to allow each participant to tell their own story with prompt questions asked as appropriate as the narratives developed. These interviews lasted between 10 and 45 minutes.

Second interviews, lasting between 10 and 62 minutes, were conducted mid-way through treatment with 32 patients and 15 caregivers. These were treated as part of an on-going story of the patients’ and caregivers’ experiences of cancer diagnosis and treatment. Participants were asked about their chemotherapy treatment and any concerns they had about the safety of their care; questions depending, in part, on the stories they had told at the first respective interview.

Post-chemotherapy 29 patients and 11 caregivers undertook a final telephone interview, this occurring within 4–6 weeks of chemotherapy cycle completion. Contemporaneous notes made during telephone interviews were transcribed immediately afterwards. These telephone interviews were between 15 and 25 minutes duration, questions again being informed in part by participants’ stories in earlier interviews. Field observations of patients and nurses interacting, including 16 of these patients on the first occasion that their chemotherapy was administered, were recorded manually within 48 hours of the observation taking place, providing ethnographic vignettes with which to contextualise the consent process (Jacobsen, 2014). These observations were between 15- and 125-minute duration.

Participants’ stories are invariably shortened rather than complete versions of events (Boje, 1991). Each offers a participant’s ‘emplotment’ (Syrjälä et al., 2009: 268) of connected sequential events related to their experiences of the consent process within the context of their treatment for cancer. Invariably through theorising, analysing and categorising their personal narratives, we become part authors of the participants’ stories; our ways of interpreting the data being influenced by our previous knowledge and experience which includes NHS nursing and social care.

**Figure 1.**
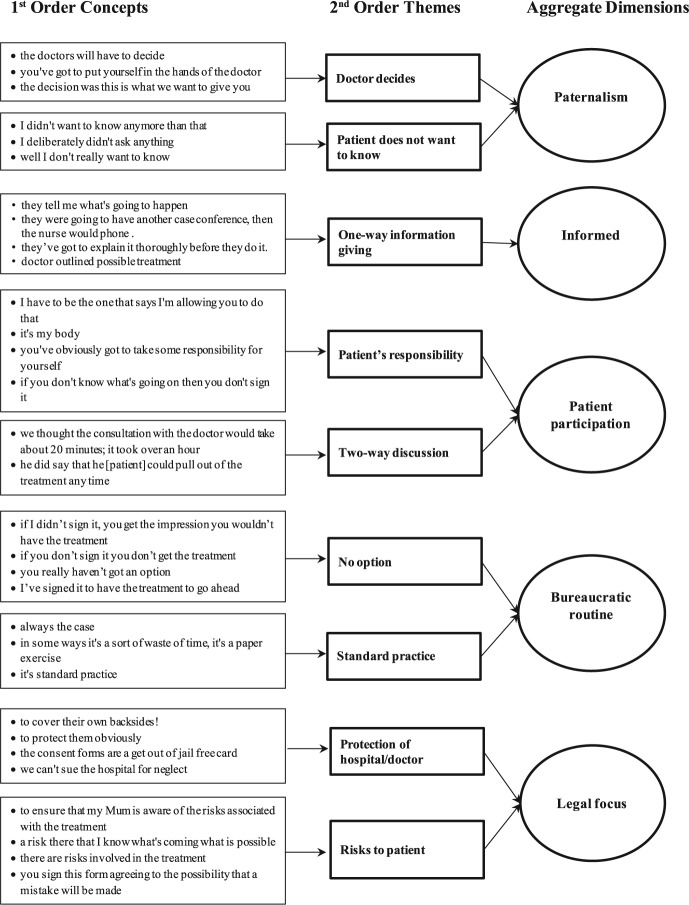
Data Structure.

Analysis of these narratives, and vignettes from field observations adopted Corley and Gioia’s (2004) grounded approach. First-order concepts within each participant’s narrative were identified initially and grouped into categories using open coding (Corbin and Strauss, 2008). Participants’ own words (‘in vivo’ codes) were used where possible providing first-order concepts such as ‘the doctors will have to decide’ and ‘the decision was this is what we want to give you’ (Figure 1). Relationships discerned between and among the first-order concepts provided the second-order themes; the two concepts referred to earlier, with others, comprising the theme ‘Doctor decides’. We undertook this process by searching for corroborating and contradictory evidence, constantly comparing accounts to select and reduce the first-order concepts to a core set of (second order) themes by merger and delimitation. Alongside this process, through debate and reference to the literature on consent to treatment and the doctor–patient relationship, possible explanatory aggregate dimensions developed (Corbin and Strauss, 2008). For example, the second-order themes ‘Doctor decides’ and ‘Patient does not want to know’, we explained by ‘Paternalism’. In the following section, we discuss our findings and examine the implications for patients’ active participation in the consent process.

## Findings

### Paternalism

The aggregate dimension of paternalism was an important finding from our analysis. Most patients in the study expected, and were content to let doctors make decisions, justifying this on the basis of doctors’ expertise:

I think the decision was this is what we want to give you. … As far as I’m concerned they tell me what’s going to happen, I consent to what’s going to happen and I sign my signature as consent to that operation … or whatever I’m having. (P32F68)

They [doctors] know better than me, so whatever they decide, the main thing is that they said they will look after me and keep me well. (P13M76)

The doctors will have to decide whether they will give me any more treatment, so all I can do is wait and see. I just have to be patient. (P18F79)

### Informed

Patients were generally disinclined to make decisions about their treatment. However, misunderstandings could arise later if they believed that doctors had not provided sufficient information regarding those treatments for which they were giving consent, or had neglected to outline fully potential alternative treatments that might be required:

I thought I was just going to have a total hysterectomy, an omentectomy but I signed for whatever else and I ended up doing that, but they [surgeons] said it was the best thing to do because they’ve taken it [cancer] all out. I’m glad they did it, I’d rather it all gone than still there. (P4F74)

Despite this patient appearing to trust the surgeons and giving consent for them to proceed as they thought necessary, post-surgery she commented ‘I didn’t realise what they were going to do’; recalling that the surgeon said, ‘come in next week and I’ll go in and have a look round’ [meaning exploratory surgery]. However, she was ‘shell shocked’ to wake up in intensive care to discover that she had been in theatre for around 10 hours because the cancer had spread further than anticipated. Two weeks following discharge from hospital during an out-patient consultation she attempted to find out what surgery had occurred:

I went to see Mr X [surgeon] and I said ‘what have you done? Did you do an extended hemicolectomy or did you do?’… and he looked at me and I said ‘it’s alright, I’m a nurse’. (P4F74)

This patient’s narrative suggests a perception that the surgeon is reluctant to provide details of the full extent of the surgery and seeks justification for her request. Guidance given to doctors states that if during a surgical procedure it becomes apparent that further lifesaving treatment is necessary but has not been consented to, the surgeon can proceed on the basis of the patient’s best interests (DH, 2009). However, the GMC (2008) cautions that where possible, the prospect of such a situation should be discussed with the patient in advance. This patient outlines how she ‘wasn’t prepared’ and commented subsequently that she now takes her son with her when she has an appointment with a surgeon, suggesting a loss of trust.

### Patient involvement

When discussing the consent process less than a third of participants mentioned patient involvement. Those who did noted that as adults, they had a responsibility to understand what was being proposed:

We’re all adults at the end of the day aren’t we so it’s an opportunity to demonstrate that they’ve [doctors] explained those risks and that we’re comfortable that it’s the best way forward. (P22F34)

I think the consent form is that they’ve explained everything to you and you know what’s going on and if you know what’s going on you should sign it and if you don’t know what’s going on then you don’t sign it. (P13M76)

They asked me if I wanted to have the chemo before the surgery; and that was a decision that they involved me in. (P5F39)

While these patients did not always wish to make decisions about their treatment, the provision of information and having the opportunity for discussion was valued, often being more positive than they and their caregivers expected:

I want to ask questions and I have found it easy to talk to them [doctors]. Some doctors they can make you feel silly when you ask questions, like they evade or don’t answer your questions. (P18F79)

I think doctors presume that people don’t want to know everything but I do. (P5F39)

We were allowed to ask questions and again we got a straightforward answer to every question. (C5M66)

The doctor explained everything to her and what was going to happen … we honestly thought the consultation would take about 20 minutes; it took over an hour, now whether that was because we were asking questions … [that was a] nice feeling, we felt that people had the time. (C7M61)

Nurses were another importance source of information for these patients and caregivers:

We had this nurse practitioner … and she was excellent, she spent a lot of time with us … and it was a two-way thing, we kept saying things so that she knew we were understanding it or maybe she corrected it if we weren’t understanding. (C5M66)

Such opportunities for further discussion with nurses appear to facilitate improved understanding and knowledge. Nurses playing a central role in providing patients and their caregivers with on-going opportunity for discussion about, and improved understanding of, their treatment (Dartey et al., 2010). Patients starting chemotherapy were provided with a booklet explaining the treatment and possible side effects. This was supported by a verbal presentation, given by a nurse to small groups of patients immediately prior to the administration of their first treatment. Our field notes record how nurses consistently checked with patients regarding their understanding of their treatment and if they had further questions as they prepared patients for the intravenous infusion of chemotherapy drugs. This is good practice, evidence suggesting that patients’ understanding can be enhanced when information is provided in various formats (Fink et al., 2010). Such involvement of health professionals other than doctors in providing treatment and advice has been acknowledged by the courts, court decisions now being applicable to all health professionals (*Montgomery v Lanarkshire Health Board*, 2015).

While clinical staff were the primary source of information, approximately one-fifth of participants also sought information from others who had experience of cancer and searched the Internet:

I attended a breast cancer forum in X [name of city] for two days and that was brilliant because you meet all the young women between the age of 20 and 45 and can talk to them about side effects or whatever. (P2F40)

I think I’m best sticking to that [information from doctors] because when I go on the Internet I do always come across things that aren’t good you know. I think they gave me everything I probably needed to know … so really all I found on the Internet was reiteration of what they had told me. (P5F39)

Nonetheless, information provided was not always what was required. For example, a woman who had attended a hospital for investigation of a breast lump said she had not been informed of the possible risks. She explained that she was feeling very uncomfortable after the procedure but did not know why. She believed this was particularly concerning because she had been told that she could drive home afterwards:

[An] unsafe experience I’ve had is when I had an arterial bleed … when they did the lymph node biopsy they hit an artery which is why I was really uncomfortable … but I had no idea, nobody told me that it was a rare complication. (P8F60).

Yet, her caregiver, an NHS consultant said that while her experience was very upsetting, she had just been unlucky. From a medical perspective, he believed that it was neither possible nor indeed desirable to alert patients to all the potential complications:

… if you did alert patients to all the very rare complications that might occur, you’d have very few people taking you up on the therapies that you’re offering. (C1M57)

Such differing perspectives highlight conflicting understanding of the type of information patients may require. For C1M57, the issue was the relative risks arising from the medical treatment. An argument that disclosure of information may make patients anxious and decline treatment is possibly paternalistic in both its aims and assumptions (Jones, 1999). However, this patient’s caregiver was not concerned about treatment (medical) risks per se; rather her concern appears to have been about the functional risks, in this case driving after a biopsy. This indicates that the reasonable doctor standard may be qualitatively different to the reasonable patient standard.

### Bureaucratic routine

Although the process of information giving appeared to meet the expectations of almost all of the patients, the actual signing of a consent form was perceived generally to be bureaucratic rather than for the patients’ benefit. Many participants described the purpose of the consent form as ‘obvious’; ‘to reassure them [doctors] really … she [wife] has to be seen to be happy about that’. (C17M56) and something that ‘you [patients] always do’ (P4F74). The patient’s signature on the form being a routine bureaucratic ‘standard procedure’ (C7M61) in the interest of the hospital; patients and carers perceiving their role in signing the form as non-negotiable ‘you have to sign to consent to any medication, that’s standard procedure’ (P3F58).

A field note illustrates how this perception can be problematic for the nurses working in the chemotherapy suite:

The chemotherapy suite ward sister said that if a patient’s chemotherapy treatment is changed or if, on a rare occasion in the out-patient clinic, the doctor forgets to ask the patient for their signature, the patient is told when they arrive for treatment that they have to wait on a doctor. This is because a consent form needs to be signed, and it has to be a doctor who does this with them. Patients often get stroppy with the nurses because they don’t see the point of this and just want to get on with the treatment without the delay involved in waiting for a doctor. (Researcher A)

Around one-quarter of the participants believed they must sign the form if their treatment was to proceed:

I’m signing my life away to say I accept it, it’s either that or die, so you really haven’t got an option have you? You either do this or you go home unsigned and die. (P23F62)

… by the time it came to the consent form it was, ‘and you need to sign the consent form for the treatment’. By which point nobody’s going to say no because if you don’t sign it you don’t get your treatment. (C1M57)

Patients appear to feel coerced into signing the consent form as a condition of receiving treatment, the process of consent doing little to encourage patients’ active participation in decisions about their treatment. None of the participants expressed concern about signing the consent form or said that they had refused to do so. Rather responses indicate they would have signed the consent form irrespective of what was written on it. Our analysis emphasises Habiba et al.’s (2004) argument that patients understand the consent process as a routine in which they are expected to enact a role that has already been scripted by doctors in the interest of doctors.

### Legal focus

Most participants assumed the primary reason for the consent form was to provide evidence they had been made aware of the risks of treatment and consequently prevent them from taking legal action against the hospital if anything went wrong. The consent form was viewed as the hospital’s ‘get out of jail free card’ (C1M57). Patients perceived the process through a medico-legal lens, signified by the need for doctors to get patients to sign the form:

To cover their own backsides! … it’s obviously so’s you can’t litigate afterwards really. (C2M37)

The get out clause in case they made a mistake. …, I’ve signed it to have the treatment to go ahead. (P15M63)

It’s to absorb [sic] them of any blame if something goes wrong. (P35M67)

Few patients believed (rightly) that the consent form was not a legal document:

It doesn’t mean there isn’t liability anywhere, at the end of the day but it’s just a consent saying I will have the treatment. They are not forcing it on me. (P25M64)

It’s my body I have to be the decider in my treatment and also as a secondary measure I would imagine it’s to safeguard the members of staff at the hospital who actually give me the treatment if there’s no evidence to show that I am willingly cooperating with it then I could sue. (P8F64)

This focus on risk echoes the legal obligation doctors have in relation to risk disclosure and, during the process of consent to treatment, emphasises the medico-legal concerns rather than to the underlying principles of patient involvement. Participants’ perceptions reflect the paternalism of the reasonable doctor standard, where the doctor speaks and the patient listens (Fovargue and Miola, 2010).

## Discussion

Doctors are advised by their regulatory bodies that the process of consent to treatment should be one of the shared decision-making, while acknowledging that some patients will not want to make clinical type decisions. In the United Kingdom, the legal position has recently shifted from one based on medical paternalism towards acknowledging that patients are active information seekers and capable of understanding medical matters (*Montgomery v Lanarkshire Health Board*, 2015). Our article explored patients’ and their caregivers’ understanding of the consent process, highlighting how their perceptions influence their active participation in decision-making and the possible medico-legal implications.

Overall, results of our study suggest that many patients do not want to make treatment decisions. Nonetheless, most patients wanted to be fully informed about their illness and the proposed treatment. Such patient involvement through provision of information appears to help them feel in control (Akkad et al., 2004).

Where patients do not expect to receive detailed information, our data suggest their information-seeking behaviour may be limited, particularly in an environment that they perceive to be paternalistic. This is important because if harm occurs, consent may be considered as invalid if it is judged that the patient was given inadequate information (*Montgomery v Lanarkshire Health Board*, 2015). Empirical evidence indicates that when doctors are required to conjecture patient preferences for information they often fail to get this correct (Strull et al., 1984; Waitzkin, 1984). Our results contributing to understanding of how patients may influence doctors’ information giving practices, as where patients do not expect to receive detailed information during medical consultation this appears to reduce their information-seeking behaviours.

From a patient perspective, our findings indicate providing adequate information appears challenging for doctors, particularly in an area of complex treatment such as cancer. Where patients believe doctors are withholding information they can lose trust. Our study also demonstrates how the reasonable doctor and the reasonable patient standards may diverge: patients may be concerned more about the functional consequences of their treatment while doctors may be more inclined to focus on the relative risks of treatment options. Such differing perspectives provide an important justification for the principle of shared decision-making (Krumholz, 2010) and are acknowledged in legal proceedings (*Montgomery v Lanarkshire Health Board*, 2015, *Reibl v Hughes*, 1980).

Heywood et al. (2010) argue such legal concerns have tainted doctors’ perceptions of consent, encouraging them to give prominence to potential risks. Few participants in our study discussed explicitly the benefits of treatment, suggesting that it may not only be doctors but patients who also give prominence to the legal aspects of the consent process. Arguably, focusing on risk may cause patients unnecessary angst and lack of appreciation of the benefits of the proposed treatment (Heywood et al., 2010). If the focus remains on risk, it has been argued that it is unlikely to improve communication between the patient and the doctor (Miola, 2009). It is also clear from our data, that the actual signing of the form may jeopardise the doctor–patient relationship, it being detrimental to trust by introducing the possibility of future legal conflict. Many patients in our study perceive the requirement to sign the consent form as bureaucratic, legalistic and solely in the interest of the hospital and its doctors. This instrumental rationality of the consent form undermines effective communication, by overlooking the agency of both individual patients and doctors and by diverting attention away from patients’ best interests (Brown, 2008).

Our study suggests that patients often perceive signing the consent form as routine and at times an inconvenience. This supports Akkad et al.’s (2004) findings that the majority of patients do not read the consent form, being content to sign an unread form on the basis of verbal information. In *Montgomery v Lanarkshire Health Board* (2015), the Supreme Court considered that patients can easily find medical information from sources such as the Internet and are therefore no longer considered by the courts as uninformed or entirely reliant upon information from doctors. Now considered in law as informed actors, patients’ attitudes towards the consent form as not being in their interests could therefore be a self-fulfilling prophecy if they are signing forms without due consideration to their contents.

## Conclusion and recommendations

Our article investigated cancer patients’ and their caregivers’ perceptions of the process of consent to treatment. Our findings suggest that much effort is being expended by doctors and nurses to enable patients to be well informed, at least in this hospital. However, the information considered important by clinical staff and patients appear to be qualitatively different; patients wish to know more about the functional consequences of their treatment while doctors focus more on the relative risks of the treatment. Our study suggests further that patients perceive signing the consent form as paternalistic, routinised and legalistic, this detracting from the information giving process and disabling their active involvement in their care.

To paraphrase Kennedy (2013), if the prevailing culture is one in which patients perceive themselves as the recipients of whatever is on offer, then consent can come to be seen as some perfunctory exercise (without which one will not gain access to treatment). Hence, the patient is ‘consented’ and the doctor can then get on with things, having had to pause as briefly as possible to tick the consent box. Incorporating information on the functional consequences of their treatment and dispensing with a box that can be ticked, that is the routine signing of the consent form, may enable patients’ more active participation both by endorsing the trust they have and need to have in their doctors and by encouraging them to engage actively and become fully involved in the information sharing process. In the words of one participant,

What’s much more important is actually sitting down and explaining to the patient what’s likely to happen and what’s not likely to happen … then sending that information in the form of a letter to the patient so that they’ve got it in writing and can read through it and come back and ask any questions. (C1M57)

As a qualitative study, the generalisations we can make are invariably theoretical, providing insights relating to cancer patients undergoing surgery within general hospitals in the United Kingdom and chemotherapy. The majority of our research participants were white Europeans and all had the capacity to consent to treatment. However, in addition to the insights offered, our findings provide the basis for further research to test our insights statistically and also extend the research to other patient groups.
